# Supplementary material for: SyNDI: synchronous network data integration framework
Source: BMC Bioinformatics. 2018 Nov 6;19:403. doi: 10.1186/s12859-018-2426-5 (PMC6219086; doi:10.1186/s12859-018-2426-5)
Supplement: Supplementary file 5 — Files of the example presented in Additional file 4. This additional file is a zip package that contains all files used in Additional file 4. Detailed descriptions of each of these files are presented in a README file included in this zip package. (GZ 30411 kb) [file 12859_2018_2426_MOESM5_ESM.gz › SyNDITools/Meme2Fimo/meme_4.9.0_4.tar.gz!/meme_4.9.0/doc/spamo.html]

SpaMo - motif spacing analysis tool


# The MEME Suite

## Motif-based sequence analysis tools

### Usage:

`spamo [options] <sequences> <primary motif> <secondary motifs>+`

### Description

SpaMo does inference of transcription factor complexes by looking for
significant spacings between binding sites.

The inputs are a set of many short sequences, a primary motif and one
or more databases of secondary motifs. It searches for the strongest
primary motif binding site and then searches in the area around it looking
for the strongest secondary motif binding site. The relative spacings of
the primary and secondary motif in all the sequences is tallied and the
probability of the close spacings happening by chance is calculated.

After all the calculations are done SpaMo outputs the non-redundant
secondary motifs in order of significance provided they had a bin that
passed the significance threshold. Similar secondary motifs are grouped
together and listed in order of significance on the secondary motif they
were redundant to. If the bin size is one then an alignment for each of
the similar secondary motifs is created.

### Inputs

#### Sequences

A FASTA formatted file containing lots of short sequences centered on
a site expected to be relevant to the primary motif. This would
typically be generated by expanding either side of a ChIP-seq peak to
obtain sequences of about 500 bases in length.

SpaMo scans the central section, excluding the margin on either edge,
for the primary motif. As the margin on each edge is excluded then if
the sequence is shorter than two times the margin plus the trimmed
length of the primary motif the sequence will always be discarded.

#### Primary Motif

A MEME formatted motif file containing a DNA motif. The primary motif
is the motif for which you are trying to find cofactors. If the file
contains more than one motif then the first will be selected by default
or another can be selected using the -primary or -primaryi options.

#### Secondary Motifs

One or more MEME formatted motif files containing DNA motifs. The
secondary motifs are tested for a significant spacing with the primary
motif which might imply they act together. If the motif databases
contain motifs which you don't wish to scan, the motifs can be filtered
based on their name by using the -inc and -exc options.

### Optional Inputs

#### Motif CISMLs

SpaMo can determine the best primary and secondary motif sites from scores in CISML files.

#### Motif Background

### Outputs

SpaMo outputs its output to files in a directory named
`spamo_out`, which it creates if necessary. You can change the
output directory using the -o or -oc options.

The main output file is named `spamo.html` and can be viewed
with a web browser. The `spamo.html` file is generated from the
`spamo.xml` file so using the xml file is recommended when
machine processing is required.

The histograms are only generated when the -eps and/or the -png options
are specified. If you are viewing the output in older web-browsers you
will need to specify the -png option so the histograms are viewable.

### Options

| Option | Parameter | Description | Default Behaviour |
| --- | --- | --- | --- |
| Input/Output | | | |
| --- | --- | --- | --- |
| -o | directory | Create the directory and write output files in it. This option is not compatible with -oc as only one output directory is allowed. | The program behaves as if `-oc spamo_out` had been specified. |
| -oc | directory | Create the directory but if it already exists allow overwriting the contents. This option is not compatible with -o as only one output directory is allowed. | The program behaves as if `-oc spamo_out` had been specified. |
| -loadcismls |  | Load CISML files to get motif position scores instead of scanning. If this flag is specified then each motif file must have a CISML file specified after it. This is not compatible with -trim as that option must modify the motifs before scanning. | Scan sequences to determine the position scores. |
| -eps |  | Output histograms in Encapsulated PostScript format which can be included in publications. This option can be used with the -png option. | Image files are not output by default as the webpage is capable of generating the graphs on demand. |
| -png |  | Output histograms in Portable Network Graphic format which is good for webpages. This option can be used with the -eps option | Image files are not output by default as the webpage is capable of generating the graphs on demand. |
| -dumpseqs |  | Write space separated values in columns, describing the motif matches used to make the histograms, to output files. The rows are initially in sequence name order but various command-line tools can be used to sort them on other values. The columns contain: column(s) \| contents; 1 \| Trimmed lowercase sequence with uppercase matches; 2 \| Position of the secondary match within the whole sequence; 3 \| Sequence fragment that the primary matched; 4 \| Strand of the primary match (+/-); 5 \| Sequence fragment that the secondary matched; 6 \| Strand of the secondary match (+/-); 7 \| Is the primary match on the same strand as the secondary (s/o); 8 \| Is the secondary match downstream or upstream (d/u); 9 \| The gap between the primary and secondary matches; 10 \| The name of the sequence; 11 \| The *p*-value of the bin containing the match, adjusted for the number of bins; If the sequence names are in Genome Browser position format (e.g., "chr5:36715616-36715623"), the following additional columns appear: \|; 12-14 \| Position of primary match in BED coordinates; 15 \| Position of primary match in Genome Browser coordinates; 16-18 \| Position of secondary match in BED coordinates; 19 \| Position of secondary match in Genome Browser coordinates | No specific match information is output. |
| -dumpsigs |  | Same as -dumpseqs, but only secondary matches in significant bins are dumped. | As in -dumpseqs. |
| Scanning | | | |
| --- | --- | --- | --- |
| -numgen | seed | Specify a number as the seed for initializing the pseudo-random number generator used in breaking scoring ties. The seed is included in the output so experiments can be repeated. If you wish to run multiple experiments with different seeds then you can use the special value 'time' (without the quotes) which sets the seed to the system clock. | A seed of 1 is used. |
| -margin | size | The distance either side of the primary motif site which makes up the region that can contain the secondary motif site. Additionally it is the minimum gap between the primary motif site and the edge of the sequence. These constraints mean that input sequences shorter than the trimmed length of the primary motif plus two times the margin size can not be used by SpaMo. | A margin of 150 is used. For an input sequence of length 500 this means the central 200 bases are scanned for the best primary motif match and then the 300 bases surrounding the best primary site are scanned for the best secondary site. |
| -bin | size | The size of the bin used to calculate the histogram and *p*-values. A bin size of 1 is recommended as it gives better output. | A bin size of 1 is used. |
| -range | size | The distance from the primary motif site for which *p*-values are calculated to include in significance tests. A small value for range may miss significant peaks but this is a trade-off as a the larger the range the more bins have to be tested leading to a larger factor used in the Bonferroni correction for multiple tests. | A range of 150 is used. |
| -shared | fraction | After the primary motif site has been selected in each sequence the sequence is trimmed to only include a region of size margin on either side of the primary motif site. This aligned and trimmed sequence is then compared with all the other sequences and the fraction of shared bases is calculated. If the fraction of shared bases is larger than this limit then one of the sequences is eliminated. To disable this feature set the shared fraction to 1. | The shared fraction is set to 0.5 which means that the trimmed, aligned sequences must share 50% or more of their bases to be declared redundant. |
| Summarizing | | | |
| --- | --- | --- | --- |
| -cutoff | *p*-value | The *p*-value cutoff for bins to be considered significant. This is the *p*-value of the Binomial Test on the number of observed secondary spacings or more falling into the given bin, adjusted for the number of bins tested. Note that the *p*-value is only calculated and tested for bins within the distance of the primary motif as specified by the option -range. | A bin *p*-value smaller than or equal to 0.05 is considered significant. |
| -evalue | *E*-value | The minimum secondary motif *E*-value for its results to be printed. For each secondary motif, this is the minimum *p*-value of all tested bins multipled by the number of secondary motifs. The *E*-value estimates the expected number of random secondary motifs that would have the given *E*-value or lower. | Results for all secondary motifs with *E*-value smaller than or equal to 10 are printed. |
| -overlap | size | To determine if two motifs are redundant the most significant bin in the tested range in each of the motifs is compared. For the motifs to be considered redundant it needs to be possible that the sites that got counted in the bin could have overlapped, and this parameter sets the minimum overlap. For a bin size larger than 1 the overlap of the bins can not be precisely calculated as the actual site positions are not stored and so the maximum possible overlap is used. | A minimum overlap of 2 is required. |
| -joint | fraction | To determine if two motifs are redundant the most significant bin in the tested range in each of the motifs is compared. The most significant bin in each motif has the list of sequence identifiers which had a primary and secondary at the correct spacing to go into that bin. To compare the motifs for redundancy this set of sequence identifiers is compared and the size of the intersection is counted. This intersection size is divided by the size of the smaller of the two sequence sets to get the joint sequence fraction. | A minimum joint sequence fraction of 0.5 is required for two motifs to be considered redundant. |
| Motif Loading | | | |
| --- | --- | --- | --- |
| -pseudo | count | The pseudocount added to loaded motifs. | A pseudocount of 0.1 is added to loaded motifs. |
| -bgfile | file | The file containing the background frequency information used in applying pseudocounts. | The frequencies of bases in the sequences are used as a background. |
| -trim | bits | Trim the edges of motifs based on the information content. The positions on the edges of the motifs with information content less than bits will not be used in scanning. This is incompatible with the -loadcismls option as the motifs must be trimmed before scoring can take place. | Positions on the edges of the motifs with information content less than or equal to 0.25 will be trimmed. |
| -primary | name | The name of the motif to select as the primary motif. This option is incompatible with -primaryi as only one primary motif can be selected. | The first motif in the file is selected. |
| -primaryi | num | The index of the motif to select as the primary motif counting from 1. This option is incompatible with -primary as only one primary motif can be selected. | The first motif in the file is selected. |
| -keepprimary |  | If the same file is specified for the primary and secondary motifs then by default the primary motif is excluded but specifying this option keeps it. | The primary motif is excluded from the secondaries if the same file is used for the primary and secondary motifs. |
| -inc | pattern | Select the motifs with names matching the pattern. The pattern can contain shell like wildcards (e.g., '\*') though they must be escaped or quoted to prevent the shell from auto-expanding them. This option may be may be repeated and all the patterns will be used. | Unless the -exc option has been specified all the motifs are used. |
| -exc | pattern | Exclude the motifs with names matching the pattern. The pattern can contain shell like wildcards (e.g., '\*') though they must be escaped or quoted to prevent the shell from auto-expanding them. This option may be may be repeated and all the patterns will be used. | Unless the -inc option has been specified all the motifs are used. |
| Miscellaneous | | | |
| --- | --- | --- | --- |
| -help |  | Print out a help message. |  |
| -verbosity | 1\|2\|3\|4\|5 | A number that regulates the verbosity level of the output information messages. If set to 1 (quiet) then it will only output error messages whereas the other extreme 5 (dump) outputs lots of mostly useless information. | The verbosity level is set to 2 (normal). |
